# Pathologic burden goes with the flow: MRI perfusion and pathologic burden in frontotemporal lobar degeneration due to tau

**DOI:** 10.1162/imag_a_00118

**Published:** 2024-03-22

**Authors:** Christopher A. Olm, Claire S. Peterson, David J. Irwin, Edward B. Lee, John Q. Trojanowski, Lauren Massimo, John A. Detre, Corey T. McMillan, James C. Gee, Murray Grossman

**Affiliations:** Penn Frontotemporal Degeneration Center, Department of Neurology, University of Pennsylvania, Philadelphia, PA, USA; Penn Image Computing & Science Laboratory, Department of Radiology, University of Pennsylvania, Philadelphia, PA, USA; Digital Neuropathology Laboratory, Department of Neurology, University of Pennsylvania, Philadelphia, PA, USA; Department of Pathology and Laboratory Medicine, University of Pennsylvania, Philadelphia, PA, USA; Center for Functional Neuroimaging, Departments of Neurology and Radiology, University of Pennsylvania, Philadelphia, PA, USA

**Keywords:** arterial spin labeling, frontotemporal lobar degeneration, cerebral blood flow, perfusion, tauopathy

## Abstract

Regional cerebral blood flow (CBF) changes quantified using arterial spin labeling (ASL) are altered in neurodegenerative disorders such as frontotemporal lobar degeneration due to tau (FTLD-tau), but the relationship between ASL CBF and pathologic burden has not been assessed. Our objective was to determine whether regional ASL CBF acquired antemortem in patients with FTLD-tau is related to pathologic burden measured at autopsy in those same regions in the same patients to directly test the imaging-pathology relationship. In this case-control study, data were acquired between 3/4/2010 and 12/16/2018. Data processing and analysis were completed in 2023. Twenty-one participants with autopsy-confirmed FTLD-tau (N = 10 women, mean[SD] age 67.9[7.56] years) along with 25 control participants (N = 15 women, age 64.7[7.53]) were recruited through the cognitive neurology clinic at the University of Pennsylvania. All participants had ASL and T1-weighted images collected antemortem. ASL images were processed to estimate CBF and T1-weighted images were processed to estimate gray matter (GM) volumes in regions corresponding to regions sampled postmortem. Digital quantification of pathologic burden was performed to find the percent area occupied (%AO) of pathologic FTLD-tau at autopsy. Regional CBF and GM volumes were both related to pathologic burden in the same regions from the same participants. Strengths of model fits of imaging measures to pathologic burden were compared. CBF in FTLD-tau and controls were compared, with results considered significant at p < 0.05 after Bonferroni correction. We found that relative to controls, FTLD-tau displayed hypoperfusion in anterior cingulate, orbitofrontal, middle frontal, and superior temporal regions, as well as angular gyrus. For patients with FTLD-tau regional CBF was significantly associated with pathologic burden (beta = -1.07, t = -4.80, p < 0.005). Models including both GM volume and CBF provided significantly better fits to pathologic burden data than single modality models (p < 0.05, Bonferroni-corrected). Our results indicate that reduced CBF measured using ASL MRI is associated with increased pathologic burden in FTLD-tau and adds complementary predictive value of pathologic burden to structural MRI.

## 1 Introduction

Frontotemporal lobar degeneration (FTLD) is a spectrum of pathology associated with early onset progressive neurodegenerative disease characterized by neuron loss associated with an accumulation of misfolded proteins. FTLD inclusions found at autopsy are considered the ground truth diagnosis. One of the two most common FTLD pathologies is FTLD due to tau (FTLD-tau). Neuropathological staging studies suggest that patients with FTLD-tau display early pathology in frontal and temporal lobes, with later pathology in parietal lobes and relative sparing of the occipital lobe (Irwin et al., 2016,^2018^;Kovacs et al., 2020). As FTLD is often a monoproteinopathy, it is an ideal disease for studying disease monitoring tools such as perfusion measured using arterial spin labeling (ASL) MRI, as there is often only one pathological substrate to consider. We examine the anatomic distribution of reduced cortical perfusion in autopsy-confirmed patients with FTLD-tau, and we relate regional perfusion during life directly to the pathologic burden found at autopsy in the same cortical regions in the same patients.

There are important practical motivations for studying the pathologic basis for reduced perfusion in FTLD spectrum disorders. Though diagnosed clinically as a variant of frontotemporal dementia (FTD) with prominent changes in behavior and executive function or language, there are no definitive*in vivo*biomarkers for diagnosing FTLD. Disease-modifying clinical treatment trials targeting FTLD spectrum pathology are in development, so*in vivo*methods of monitoring treatment efficacy related to the gold standard pathologic burden are of great interest. MRI offers repeatable, widely available, and non-invasive candidate markers. Anatomic distributions of structural gray matter (GM) atrophy measured using T1-weighted MRI have been associated with pathologic burden found at autopsy in FTLD, but MRI atrophy is rarely directly compared to pathology in specific cortical regions (Burke et al., 2022;Giannini, Xie, McMillan, et al., 2019;Irwin et al., 2016,^2018^;Seeley et al., 2007;Whitwell et al., 2005). Yet, GM atrophy may be a relatively late event in the progression of regional disease as it reflects parenchymal loss. Arterial spin labeling (ASL) is a non-invasive MRI measure of regional brain perfusion that is quantified as cerebral blood flow (CBF; units of mL/100g/min), performs similarly to PET imaging for diagnostic utility without ionizing radiation (Ssali et al., 2022;Tosun et al., 2016), and is readily combined with structural MRI acquired in the same imaging session. ASL measures of perfusion are potentially sensitive to earlier regional disease than structural measures since perfusion may reflect reduced function prior to neuronal death in FTLD (Dopper et al., 2016;Olm et al., 2016). Hypoperfusion identified by ASL has been associated with regions that typically display high pathologic burden in FTLD (Hu et al., 2010), though this has not been tested directly by relating*in vivo*and postmortem measurements from the same regions in the same individuals, as we do here.

We hypothesized that regional CBF estimates derived from*in vivo*ASL imaging would be related to pathologic burden found at autopsy in the same regions in a group of patients with FTLD-tau. We also examined the potentially complementary role of structural MRI in predicting pathological findings. In doing so, we provide a template for future studies evaluating imaging markers against a pathological gold standard.

## 2 Methods

### 2.1 Participants

All participants underwent an informed consent procedure approved by the Institutional Review Board at the University of Pennsylvania in accordance with the Declaration of Helsinki. Participants were recruited through the cognitive neurology clinic and the Penn Frontotemporal Degeneration Center at the University of Pennsylvania. We used the Penn Integrated Neurodegenerative Disease Database (Toledo et al., 2014) to identify 24 patients with autopsy-confirmed FTLD-tau pathology and 25 age- and education-matched healthy controls with at least one antemortem high-resolution T1-weighted image and one pseudo-continuous ASL (pCASL) image acquired in the same imaging session. Experienced cognitive neurologists (M.G., D.J.I., L.M.) diagnosed patients clinically with published criteria for phenotypes confirmed at a weekly multidisciplinary consensus meeting, and patients were only recruited for research with a Fazekas score of 0 or 1. At autopsy, no “micro,” “medium,” or “large” infarcts were noted in any participant included in the present study. Two patients were excluded for lack of available regional digital neuropathology. One participant was excluded due to outlying excessive motion as determined using a cut-off of mean framewise displacement (FD) > 1 mm during ASL acquisition. Each participant had at least four regions sampled at autopsy (mean samples per participant = 8.14 +- 3.10 (SD), range 4:15) and each sampled region had a corresponding CBF estimate (see below for description of CBF estimation). Please seeTable 1for a summary of demographic information. For a summary of clinical phenotype and FTLD-tau subtype, please refer toTable 2. Expert pathologists (E.B.L., J.Q.T.) used published criteria (Toledo et al., 2014) to confirm primary pathological diagnosis of FTLD-tau at the time of autopsy. When multiple MRI sessions were available for an individual, the highest quality ASL was selected for analysis (assessed by visual inspection of CBF map).

**Table 1.** Demographic characteristics of participants.

	Controls	FTLD-tau
N (female)	25 (15)	21 (10)
Age at MRI (SD; years)	64.7 (7.53)	67.9 (7.56)
MMSE at MRI (SD) *	29.4 (0.63)	23.2 (5.34)
Education (SD; years)	16.1 (2.42)	15.5 (2.34)
Disease duration at MRI (SD; years)	N/A	5.29 (2.88)
Time from MRI to autopsy (SD; years)	N/A	2.42 (1.46)
Mean FD (SD; mm)	0.305 (0.180)	0.329 (0.192)

There was no significant difference (p < 0.05) between patients with FTLD-tau and controls for sex ratio of males to females, age at MRI, years of education, or motion during arterial spin labeling (ASL) acquisition as estimated using mean framewise displacement (FD).

SD = standard deviation, FTLD-tau = frontotemporal lobar degeneration due to tau, MMSE = Mini mental state examination at time of imaging; * = significant difference between controls and FTLD-tau by t-test (p < 0.05).

**Table 2.** Clinical and pathological details.

		Clinical phenotype						
		Behavioral variant frontotemporal dementia	Corticobasal syndrome	Dementia with Lewy bodies	Mild cognitive impairment	Non-fluent/agrammatic primary progressive aphasia	Progressive supranuclear palsy	Semantic variant primary progressive aphasia
Pathologic diagnosis	Corticobasal degeneration	—	1	—	—	2	1	—
	Pick’s disease	3	1	—	—	—	—	1
	Progressive supranuclear palsy	2	3	1	1	—	4	—
	Tauopathy unclassifiable/MAPT	1	—	—	—	—	—	—

Shown are the number of participants with each pathological subtype of FTLD-tau and primary clinical phenotype at presentation.

### 2.2 Image processing

#### 2.2.1 ASL acquisition and preprocessing

All control and patient ASL images were acquired using the same, spin echo pCASL sequence with 40 label control pairs and voxel dimensions of 2.5 x 2.5 x 5.0 mm^3^with a 1 mm slice gap, a 1500 ms post labeling delay and labeling duration, and a 0.0625s slice time, as previously described (Olm et al., 2016). The open source, publicly available ASLPrep 0.2.8 was used to process ASL data (https://github.com/PennLINC/aslprep) (Adebimpe et al., 2022). ASL is acquired as time series so we corrected for motion and other noise confounds, and then used Bayesian Inference for Arterial Spin Labeling (BASIL) to estimate CBF (Chappell et al., 2009). Partial volume correction (PVC) may account for the relatively large voxel sizes in ASL data that often include different tissues that have different characteristic CBF, namely GM, white matter, and CSF which has CBF = 0 (Chappell et al., 2021;Meltzer et al., 1990). So, we additionally applied PVC at the voxel level using the previously described extension of BASIL (Chappell et al., 2011). However, when analyzing CBF with and without PVC, results remained qualitatively similar (seeeResults in Supplement) so we present results without PVC. For more details on ASL processing, seeeMethods in Supplementand ASLPrep documentation (https://aslprep.readthedocs.io/en/latest/index.html). Please seeFigure 1for an example of preprocessed ASL data. All preprocessed ASL images were visually inspected by an experienced scientist (C.A.O.) for quality before inclusion in the study. After preprocessing, mean CBF is calculated in ROIs that correspond to regions sampled at autopsy, as described insection 2.2.2, below.

**Fig. 1.**
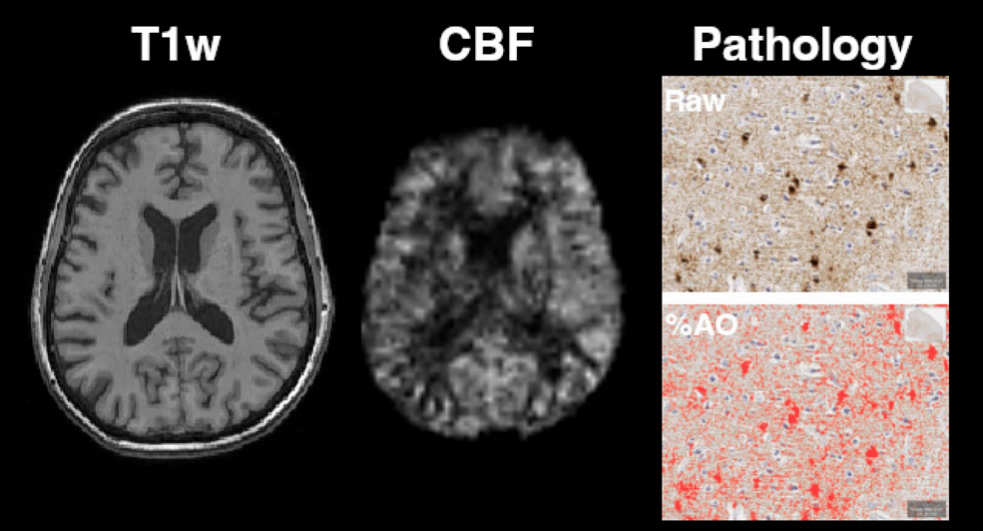
Examples of images. On the left is a structural T1-weighted image (T1w) for one participant with Pick’s disease. In the middle is a processed arterial spin labeling image, showing the voxel-wise cerebral blood flow (CBF) map from the same imaging session as the T1w. On the right is an example of the histopathology from the middle frontal cortex of the same participant, with a raw AT8-stained image on top and the corresponding percent area occupied (%AO) derived from quantitative digital histopathology below that.

#### 2.2.2 T1-weighted acquisition and preprocessing

All participant T1-weighted MRI data were acquired using the same high resolution MPRAGE sequence with TR = 1620 ms, TE = 3.09 ms, inversion time = 950 ms, flip angle = 15°, 192 x 256 matrix, and 0.98 x 0.98 x 1.0 mm^3^voxels and were processed using antsCorticalThickness.sh (Tustison et al., 2014). The pipeline uses Advanced Normalization Tools (ANTs) to perform N4 bias correction (Tustison et al., 2010), diffeomorphic registration from native space to template space (Avants, Tustison, Song, et al., 2011), and 6-tissue class (cortex, deep GM, white matter, CSF, brainstem, and cerebellum) brain segmentation (Avants, Tustison, Wu, et al., 2011). We used data from the Open Access Series of Imaging Studies (Marcus et al., 2007) to generate the local template. Brain segmentation quality was visually inspected by an expert rater (C.A.O.) for all participants before inclusion in the study. To determine regional GM volumes, we used the cortical subset of the Lausanne parcellation with 250 labels per hemisphere (Hagmann et al., 2008), generated for our local template using easy_lausanne (https://github.com/mattcieslak/easy_lausanne) (Daducci et al., 2012). The template parcellation was spatially normalized to each participant’s native T1 space using the template-to-native T1 warps generated by ANTs and was masked by the participant’s cortex. Lausanne regions at the 250 scale are too small to be reasonable regions of interest (ROIs) for the relatively large voxels in ASL imaging. Furthermore, to analyze antemortem MRI measures that correspond to regions sampled at autopsy, a team of neuroimaging experts, neuropathologists, and neurologists convened to select and merge ROIs of the Lausanne 250 parcellation together to most closely reflect regions sampled at autopsy to foster imaging-pathological analyses as previously described (Spotorno et al., 2020).

### 2.3 W-score calculation

To account for known relationships between regional CBF (mL/g/min) and age (years), and associations between GM volumes (mm^3^) and age and intracranial volume (ICV), data from the patient-matched healthy aging control participants described above were used to create w-scores for CBF and GM volumes in each ROI for each participant. W-scores are effectively z-scores with covariates of no interest removed using a linear regression model. Analyzing w-scores also allows for reasonable statistical comparisons of GM volumes and CBF.

### 2.4 Pathologic burden

Hemisphere for study was sampled at random at autopsy according to standardized NIA/AA diagnostic guidelines, as modified in (Hyman et al., 2012). We did not have sufficient power to rigorously investigate associations for each hemisphere independently, so we collapsed all data onto one hemisphere. Bilateral sampling was available for a subset of patients (N = 7) so to avoid over-weighting these participants in our statistical analysis their hemisphere data were averaged together. The available sampled regions included the anterior cingulate (ACC), superior/middle temporal gyrus (STG), orbitofrontal cortex (OFC), middle frontal cortex (MFC), angular gyrus (ANG), precentral gyrus (PrecG), dorsolateral prefrontal cortex (DLPFC), inferior frontal gyrus (IFG), inferior parietal gyrus (IPFG), superior parietal lobe (SPL), anterior insula, posterior cingulate cortex (PCC), temporal pole (TempPole), ventrolateral temporal cortex (VLT), and calcarine cortex. The number of samples from each region in each hemisphere is shown in eTable 1. Tissue was fixed in 10% formalin overnight with standard procedures at autopsy as described (Toledo et al., 2014). Tissue examined in this study was immunostained for mature tau (AT8; Invitrogen) (Mercken et al., 1992) using standardized procedures (Giannini, Xie, Peterson, et al., 2019) in the Penn Digital Neuropathology lab. Whole slides were imaged with 20x magnification with an Aperio AT2 (Leica Biosystem, Wetzlar, Germany) (Giannini, Xie, McMillan, et al., 2019;Irwin et al., 2016) in the Penn Digital Pathology Lab. QuPath 0.3.0 (Bankhead et al., 2017) was used to measure the percent area occupied (%AO) of FTLD-tau inclusions in GM in each slide (Giannini, Xie, Peterson, et al., 2019;Irwin et al., 2016). To account for differences in morphology between subtypes of FTLD-tau, each subtype (namely, progressive supranuclear palsy, corticobasal degeneration, Pick’s disease, and unclassified tauopathy associated with a mutation in the*MAPT*gene) was min-max normalized and then we took the natural log of these values, as previously reported (Giannini, Xie, McMillan, et al., 2019), to improve normality as determined using density and quantile-quantile plots to facilitate parametric statistics.

### 2.5 Relating CBF to pathologic burden

#### 2.5.1 CBF comparisons between controls and patients with FTLD-tau

To determine whether patients with FTLD-tau displayed abnormal CBF, we performed two-sided two-sample t-tests of the CBF w-scores to compare patients to controls at each ROI with more than 4 patient samples, considering results significant at p < 0.05 (Bonferroni-corrected for number of ROIs analyzed, N = 12). To allow for direct consideration of hypoperfusion in patients relative to controls and pathologic burden, we limited analysis to regions that were sampled at autopsy in patients. Similar analysis was performed to compare the regional GM volumes between patients and controls, again considering results significant at p < 0.05 (Bonferroni-corrected).

#### 2.5.2 Regional CBF and pathologic burden

We used linear mixed-effects models (LMMs) to relate regional CBF (fixed effect) to regional pathologic burden quantified as ln (normalized (%AO)). We thus determined whether*in vivo*perfusion predicts pathologic burden. Inter-subject variance in labeling efficiency likely influences differences in global CBF between individuals (as noted above, each participant has 4 or more regions included in the analysis), so the participant was included as a random effect of no interest. Not all patients have had a comparable duration between their MRI and death and this duration likely contributes to any observed relationship between perfusion estimates and pathologic burden, so we included this interval (in years) as a fixed effect of no interest. For each model, we used the lme4 package in R to perform the LMM (Bates et al., 2015), and results were considered significant at p < 0.05. We set restricted maximum likelihood (REML) to false to allow for use of maximum likelihood tests to compare models. To determine if any relationship between perfusion and pathologic burden was mediated by clinical phenotype, analyses were rerun with primary clinical phenotype as an additional fixed effect. Similarly, an analysis was run with pathological subtype as an additional fixed effect.

#### 2.5.3 Accounting for structure

There are known relationships between observed GM structure and CBF estimates (Chappell et al., 2009,^2011^), and between GM structure and %AO (Giannini, Xie, McMillan, et al., 2019;Irwin et al., 2016,^2018^). Thus, accounting for structure is necessary to provide a more complete picture of the complex relationships between pathologic burden, GM structure, and GM function. As such, analyses were run with both CBF and volume as fixed effects. We used Vuong’s closeness tests (Vuong, 1989) to compare pairs of models with and without volume included as a fixed effect to determine whether (1) models with CBF and volumes are a better fit than models with just CBF, and (2) models with CBF and volumes are a better fit than the model with just volumes, considering results significant at p < 0.05.

## 3 Results

### 3.1 Patient-to-control CBF comparisons

We compared mean CBF in each of the ROIs sampled at autopsy to determine whether CBF was different between patients with FTLD-tau and controls. As shown inFigure 2, of the regions examined at autopsy, ACC, OFC, MFC, STG, and ANG displayed significant hypoperfusion for each CBF estimate in FTLD-tau relative to controls (p < 0.05, Bonferroni-corrected). There were no regions of significant hyperperfusion.

**Fig. 2.**
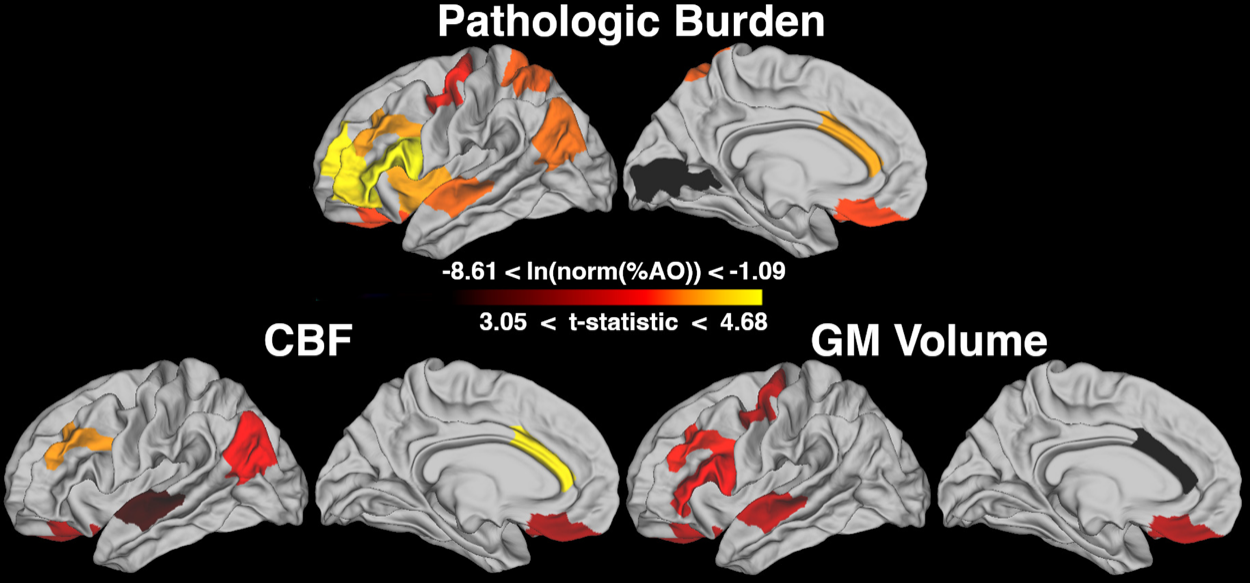
Comparison of cerebral blood flow (CBF) and gray matter (GM) volumes between patients with frontotemporal lobar degeneration due to tau (FTLD-tau) and controls. Mean postmortem pathologic burden of FTLD-tau inclusions (natural log of normalized percent area occupied; ln (norm(%AO))) in all regions with > 4 samples are shown in the top panel. The bottom panels are comparisons of*in vivo*MRI measures between patients with FTLD-tau and controls with differences considered significant at p < 0.05, after Bonferroni correction for multiple comparisons, again in all regions with > 4 samples. The color scale bar represents ln(norm(%AO)) for pathologic burden, and t-statistics for CBF and volumes (with only regions reaching significance shown).

### 3.2 Perfusion is related to pathologic burden

Next, we tested the relationship between CBF and pathologic burden. As shown inFigure 3andTable 3CBF demonstrated a significant relationship (p < 0.005, Bonferroni-corrected) between CBF and pathologic burden. Results were similar when accounting for clinical phenotype and when accounting for FTLD-tau subtype; and some clinical phenotype and FTLD-tau subtype groups were too small (N = 1) to reasonably interpret so results without clinical phenotype and without FTLD-tau subtype included in the model are examined below. As shown in eResults, CBF estimated using PVC shows a similar overall pattern. We also repeated our regression analysis without participant as a random factor and again found that CBF was a significant predictor pathologic burden (p < 0.005; data not shown).

**Fig. 3.**
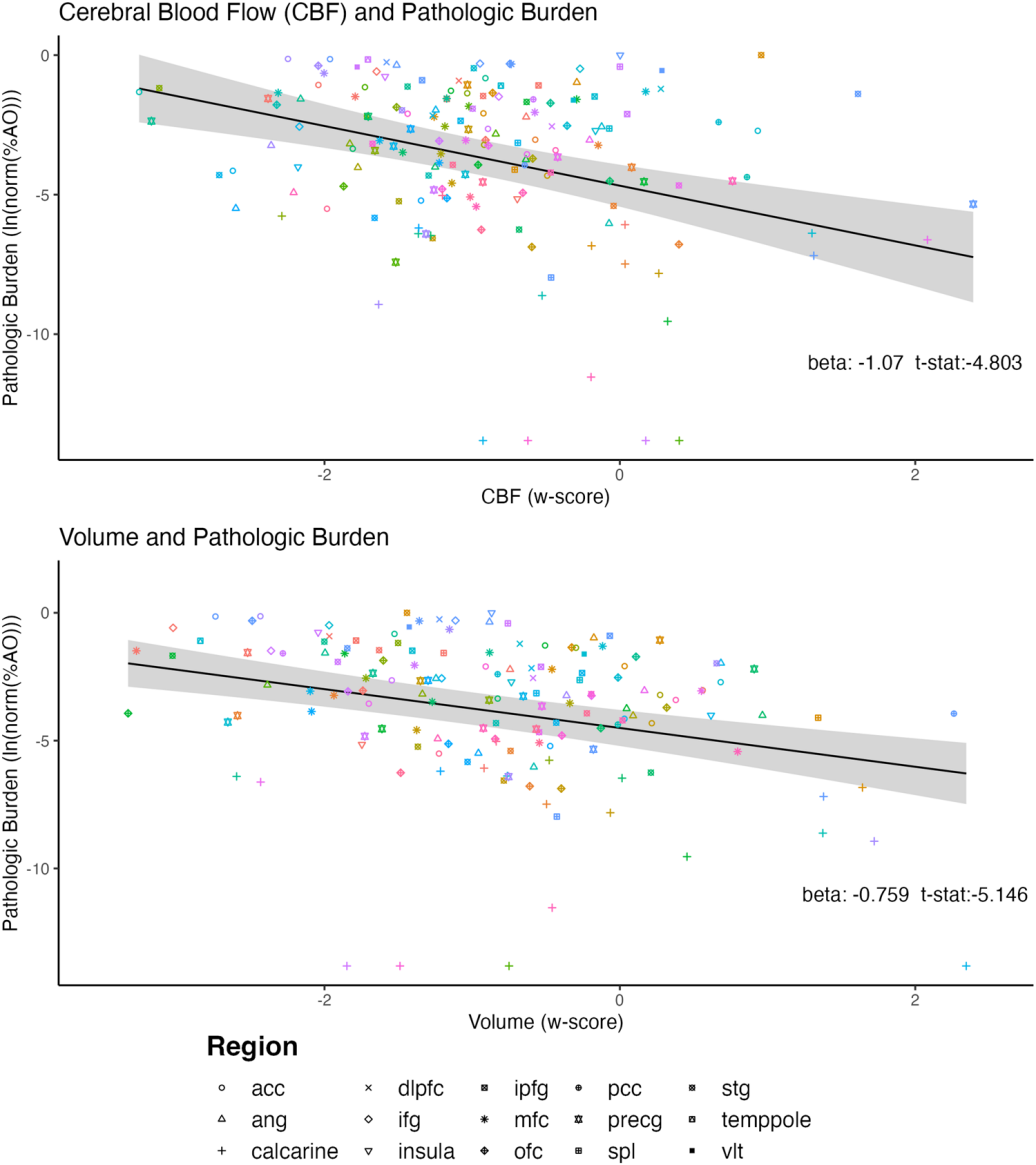
Cerebral blood flow (CBF) and pathologic burden. Cerebral blood flow (CBF) has a significant relationship with pathologic burden (p < 0.005, after Bonferroni correction for multiple comparisons). Each point represents measures from one region, and each color represents one participant. The trend line represents best fit of the w-Scores of CBF as a fixed effect on the natural log of the normalized percent area occupied data (ln(norm(%AO))) in models with MRI-to-autopsy interval and participant as additional effects in the linear mixed-effects model, with the grey band representing the 95% confidence interval of the model fit. The beta and t-statistic are reported for the fixed effect of CBF. Shape of each point represents region (though this was not accounted for statistically). Abbreviations: cerebral blood flow (CBF), anterior cingulate (acc), superior/middle temporal gyrus (stg), orbitofrontal cortex (ofc), middle frontal cortex (mfc), calcarine cortex, angular gyrus (ang), precentral gyrus (precg), dorsolateral prefrontal cortex (dlpfc), inferior frontal gyrus (ifg), inferior parietal gyrus (ipfg), superior parietal lobe (spl), insula, posterior cingulate cortex (pcc), temporal pole (temppole), and ventrolateral temporal cortex (vlt)

**Table 3.** Statistical model details.

Dependent variable (R-squared)		Beta	Standard error	t-statistic	p-value
Mean CBF (r ^2^ = 0.271)	*(Intercept)*	−5.715	0.638	−8.956	0
	*wvalue*	−1.070	0.223	−4.803	3e-06
	*MRItoDeath*	0.389	0.198	1.968	0.063
Mean CBF, no cleaning (r ^2^ = 0.181)	*(Intercept)*	−5.195	0.584	−8.900	0
	*wvalue*	−1.010	0.286	−3.538	0.001
	*MRItoDeath*	0.347	0.182	1.908	0.071
PVC CBF (r ^2^ = 0.304)	*(Intercept)*	−6.001	0.678	−8.851	0
	*wvalue*	−1.188	0.236	−5.043	1e-06
	*MRItoDeath*	0.437	0.207	2.107	0.048
Volume (r ^2^ = 0.286)	*(Intercept)*	−4.871	0.578	−8.422	0
	*wvalue*	−0.759	0.147	−5.146	1e-06
	*MRItoDeath*	0.138	0.196	0.701	0.492

Linear mixed-effects models were used to evaluate ability of*in vivo*MRI metrics: gray matter volume and cerebral blood flow (CBF), as well as partial volume corrected (PVC) CBF, to predict pathologic burden at autopsy in the same region.

### 3.3 Perfusion is related to pathologic burden after accounting for structure

We tested the relationships between CBF and GM volumes and pathologic burden and found that both CBF and GM volume were significant predictors of pathologic burden (p < 0.005, Bonferroni-corrected). The GM volume + CBF model provided a significantly better fit than the model with only GM volume (p < 0.05, Bonferroni-corrected). The GM volume + CBF model was also a significantly better fit than the model with only CBF (p < 0.0005, Bonferroni-corrected).

## 4 Discussion

We demonstrated that CBF measured*in vivo*using pCASL MRI is associated with “gold standard” pathologic burden in the same regions measured at autopsy in patients with FTLD-tau. Brain regions displaying relatively more pathologic burden in patients with FTLD-tau at autopsy tended to display greater hypoperfusion relative to controls*in vivo*. This finding supports the notion that ASL CBF is sensitive to neuropathological changes in FTLD-tau. When adding GM volume derived from structural MRI to CBF in the models predicting pathological burden, the prediction was improved. This demonstrates that ASL CBF measures are complementary to structural volumetry and can be combined to improve estimates of pathological burden*in vivo*.

Similar to reports of regional pathologic burden at autopsy, ASL imaging of patients with FTLD measured*in vivo*has generally shown hypoperfusion in frontal and temporal regions (Binnewijzend et al., 2014;Du et al., 2006;Hu et al., 2010;Tosun et al., 2012), though pathologic burden is also found in parietal regions in FTLD-tau (Irwin et al., 2016). However, we are unaware of previous evidence directly relating ASL imaging findings to pathologic burden in the same patients. Here, we observed hypoperfusion in ACC, echoing findings of early pathologic inclusions found at autopsy in this region in FTLD-tau (Irwin et al., 2016;Seeley et al., 2012). We also found hypoperfusion in other regions accumulating pathologic burden relatively early in the disease course: OFC, SMT, and MFC (Irwin et al., 2016). As shown here, ANG has previously shown evidence of early FTLD-tau at autopsy without structural changes detected*in vivo*using structural T1 images (Irwin et al., 2016), yet we did find evidence of hypoperfusion in this region. This would be consistent with the hypothesis that ASL may detect reduced neural function occurring prior to overt neurodegeneration, while volume loss associated with cortical thinning may be sensitive only to end-stage conditions such as neuronal death. Alternatively, it is possible that disease present in frontal regions connected to ANG through the superior longitudinal fasciculus contributes to diaschisis-like hypoperfusion in ANG (Baron et al., 1981;Irwin et al., 2016). In contrast, we also found apparent volume loss in PrecG and IFG without significant hypoperfusion at the conservative Bonferroni-corrected threshold used for reporting (though they do reach significance without correction for multiple comparisons). Properties of imaging acquisition, such as larger voxels in ASL relative to T1-weighted imaging, may reduce the sensitivity of ASL imaging to some subtle signs of disease seen in T1-weighted volumetric imaging. Alternatively, this may indicate a region where some atrophy has occurred and the remaining neural population in that region is recruited to maintain function. Though not found in the present study, hyperperfusion is sometimes observed in FTLD and other neurodegenerative diseases (Ferraro et al., 2018;Roquet et al., 2016), potentially as a compensatory mechanism where other brain regions are recruited to compensate for some regions showing early signs of disease (Cabeza et al., 2002;Ferraro et al., 2018;Hu et al., 2010;Roquet et al., 2016), or possibly as a result of neuroinflammation. Though phenotype was not found to significantly alter the relationship between CBF and pathologic burden in the (limited) analysis presented, clinical phenotype likely influences cortical atrophy and pathology burden (Giannini et al., 2021;Kovacs et al., 2020;Whitwell et al., 2020) so future work should more closely examine regional differences in perfusion and pathology across clinical phenotypes in adequately powered samples. Furthermore, our preliminary analysis did not find any significant effect of FTLD-tau subtype on the models, but subtype may also affect pathologic burden and distribution. Therefore, future work with larger samples of these subtypes and examination of the somewhat rarer globular glial pathology is necessary to better understand MRI-pathology relationships in FTLD-tau. This is of particular interest as tau-PET ligands are in development, though binding of tau-PET with different FTLD-tau subtypes is still relatively poorly understood (Levy et al., 2022;Tsai et al., 2019), so ASL may be a sensitive but less specific candidate marker of regional pathologic burden.

The model including both GM volumes and CBF was the best model of pathologic burden, perhaps reflecting that perfusion and structural imaging are sensitive to different processes, or different stages of disease, and provide complementary information. Past work has demonstrated a relationship between pathologic burden and GM structure in FTLD-tau (Burke et al., 2022;Giannini, Xie, McMillan, et al., 2019;Irwin et al., 2016,^2018^), and the existence of an interrelationship between brain function and brain structure is well established (Chappell et al., 2021;Du et al., 2006;Meltzer et al., 1990;Olm et al., 2016;Tosun et al., 2012) so atrophy and partial volume effects (PVE) may influence the demonstrated CBF-pathologic burden relationship. However, our findings that CBF decreases as pathologic burden increases both with and without PVC, and that CBF improves model fit relative to GM volume alone, suggests each MRI modality contributes unique information about the pathologic burden. The particular effects they capture may differ across different brain regions due to different local morphometry or cellular distribution (Kandel et al., 2015), or the relative maturity of the pathological protein being measured (Augustinack et al., 2002). Similar to past studies supporting multimodal MRI analysis (Tosun et al., 2012;Zhang et al., 2011), our results demonstrate that multimodal MRI data may better identify diseased brain regions in FTLD-tau and have the potential to improve characterization and understanding of brain changes related to pathology in dementia possibly even before symptoms are detectable (Dopper et al., 2016). This also highlights the utility of collecting ASL and T1-weighted MRI in the same session, as metabolic imaging such as FDG-PET has been shown to be highly correlated with perfusion derived from ASL in FTD and other neurodegenerative diseases, but requires the use of ionizing radiation (Ssali et al., 2022;Tosun et al., 2016) and multiple scanning sessions which increases burden on patients, their caregivers, and medical facilities.

Our study uniquely integrates multimodal MRI with gold-standard quantitative histopathology data in the same patients in the same regions and our results provide evidence that CBF measured*in vivo*using ASL is associated with pathologic burden measured at autopsy. Yet, our study is not without its limitations. First, our sample is relatively small. Moreover, though we normalized by subtype FTLD-tau is a pathologically heterogeneous disease. Furthermore, FTLD-tau is but one pathologic inclusion; additional studies are needed to determine whether similar antemortem-imaging-to-postmortem-pathology relationships are present in other pathologies implicated in FTD, such as FTLD due to TDP and FUS and ALS due to SOD1. Although we have uniquely dense sampling of pathologic burden for many patients, patients with FTLD may not display symmetric pathologic burden, such as a preponderance of primary progressive aphasia cases displaying more pathology in the left hemisphere than the right, and behavioral variant frontotemporal dementia tending to demonstrate heterogenous patterns of interhemispheric distributions of pathology (Irwin et al., 2018), so more bilateral sampling of more regions and other methods to interrogate larger brain structures should be performed in the future. Though AT8 is a well-validated marker of FTLD-tau, it is only one measure of disease and may not capture complex interactions of gliosis and neuron loss, so additional markers of neurodegeneration should be examined as digital pathology approaches are developed and validated. FTLD-tau is relatively rare and a strength of this work is the well-characterized autopsy cases collected over many years, yet this means that the ASL imaging sequence used for this study is no longer considered state-of-the-art (Alsop et al., 2015;Vidorreta et al., 2013). Although pCASL remains the preferred ASL labeling method as a good balance between high signal-to-noise and high labeling efficiency (Alsop et al., 2015), the spin-echo echoplanar readout has a previously reported cyclic lipid-shift artifact that relies upon pair-wise averaging to remove (or at least minimize) the artifact (Olm et al., 2016). ASL studies using background-suppressed 3D readouts provide much better sensitivity (Vidorreta et al., 2013) and are now preferred (Alsop et al., 2015). BASIL is dependent upon accurate processing of structural T1-weighted images so future work should also assure multimodality processing is optimized for these applications. Though results that accounted for PVCs were similar to those without, PVEs can obscure changes in a single compartment and future work should rigorously investigate their effect on imaging marker-pathologic burden relationships (Chappell et al., 2021;Greve et al., 2016;Meltzer et al., 1990), and explore alternative methods to account for structure-function relationships (Kandel et al., 2015). Future work should also examine CBF in neurodegenerative disease in the context of additional vascular risk factors, as well as additional imaging such as FLAIR that may show signs of vascular disease that may be important to consider when analyzing ASL data, and using multi-PLD ASL acquisitions that may better capture effects of vascular disease.

## 5 Conclusion

We have demonstrated that perfusion measured using ASL is predictive of pathologic burden at autopsy in FTLD-tau. Greater hypoperfusion was associated with greater pathologic burden at autopsy. Furthermore, models predicting pathologic burden including both CBF and GM volume are improved relative to either modality alone, suggesting that CBF and GM structure capture unique aspects of pathologic burden and disease in FTLD-tau.

## Supplementary Material

Supplementary Material

## Data Availability

Deidentified data can be shared upon reasonable request.
