# Supplementary material for: Pathologic burden goes with the flow: MRI perfusion and pathologic burden in frontotemporal lobar degeneration due to tau
Source: Imaging Neurosci (Camb). 2024 Mar 22;2:imag-2-00118. doi: 10.1162/imag_a_00118 (PMC12247599; doi:10.1162/imag_a_00118)
Supplement: Supplementary Material [file imag_a_00118-supp.pdf]

Supplement

eMethods: ASL Acquisition and Preprocessing
eTable: Number of pathologic burden samples per region.
eResults: Results comparing BASIL CBF and BASIL CBF with PVC
eReferences

eMethods
ASL Acquisition and Preprocessing:
ASL MRI data were acquired using pCASL with a spin echo echo-planar imaging readout with 40 label-control pairs, TR=4300ms, TE=20ms, 2.5x2.5mm^2 in-plane resolution, 5mm thick slices, 1mm slice gap, 1500ms labeling duration, 1500ms post-labeling delay, and a 96x96 matrix. All images passed a visual quality control. pCASL MRI images were preprocessed using ASLPrep 0.2.8(Adebimpe et al., 2022), which is based on Nipype 1.7.0(Gorgolewski et al., 2011). Many internal operations of ASLPrep use Nilearn 0.8.1(Abraham et al., 2014), NumPy(Harris et al., 2020), and SciPy(Virtanen et al., 2020). We briefly describe the pipeline below. For more details of the pipeline, see the ASLPrep documentation (https://aslprep.readthedocs.io/en/latest/index.html).

First, the middle volume of the ASL timeseries was selected as the reference volume and brain extracted using Nipype's custom brain extraction workflow. ASL runs were slice-time corrected using 3dTshift from AFNI(Cox & Hyde, 1997). Head-motion parameters were estimated using FSL's mcflirt(Jenkinson et al., 2002). ASLPrep co-registered the ASL reference to the T1-weighted image using FSL's flirt(Jenkinson & Smith, 2001) with 6 degrees of freedom, and several confounding factors were calculated for each ASL run, including framewise displacement (FD)(Power et al., 2014).

The CBF was quantified using three processing strategies (see descriptions of each method, below) as implemented in the ASLPrep processing suite(Adebimpe et al., 2022). After processing, each CBF map was transformed to T1-grid-space using antsApplyTransforms configured with Lanczos interpolation to minimize the smoothing effects of other kernels(Lanczos, 1964).

To associate regional CBF with regional pathologic burden, CBF was summarized using regions of interest (ROIs) corresponding to regions sampled at autopsy. An interdisciplinary team of experts in *in vivo* MRI and postmortem neuropathology collaborated to select ROIs from the 250 scale Lausanne parcellation that best corresponded to regions typically sampled at autopsy, as previously described(Burke et al., 2022). Before calculating mean CBF in each ROI, in-house scripts were used to remove non-physiological noise, namely non-physiological CBF values below 0 and voxels that were intensity-clipped at the scanner due to suboptimal scanner gain calibration. The voxels with clipped or negative values, which were present in each raw dataset, were excluded from mean CBF calculations.

ASL, including pCASL, is typically acquired as a time series of label-control pairs in the same session as a T1-weighted structural image, which allows for methods to clean/estimate noise when estimating CBF. BASIL, implemented in FSL 6.0.3, estimates CBF based upon all label-control difference values in the same region, as well as the GM, WM, and CSF probabilities generated from the T1-weighted image(Chappell et al., 2009). Additionally, BASIL uses priors based upon known physiological information: in particular, it forces CBF in CSF to zero and removes values that are calculated as less than zero(Chappell et al., 2009). Furthermore, BASIL models out Gaussian noise when estimating the voxel-wise CBF(Chappell et al., 2009).

As mentioned above, PVEs and PVC are important to consider in studies of neurodegenerative disease, such as FTLD-tau, when atrophy is present and potentially confounding CBF measures. Thus, an extension of BASIL implements a form of PVC, based upon tissue fractions of GM, WM, and CSF from segmentation of T1-weighted images, to account for the PVEs whereby an image is created of the GM contributions to CBF, creating a "BASIL+PVC" CBF estimate(Chappell et al., 2011).

An alternative to the above-described methods for estimating CBF is a "Simple" averaging together of all label-control pairs using a general kinetic model(Buxton et al., 1998), after motion-correction and confound removal as recommended in(Alsop et al., 2015).

	bvFTD	CBS	DLB	MCI	naPPA	PSP	svPPA	Total
ACC	6	5	1	1	2	5	1	21
STC	6	5	1	1	2	5	1	21
MFC	6	5	1	1	2	4	1	20
OFC	5	5	1	1	2	5	1	20
Calcarine	6	5	1	1	2	4	1	20
PrecG	5	5	1	1	2	3	1	18
ANG	5	3	1	1	2	3	1	16
SPL	2	0	0	1	2	0	1	6
aINS	2	0	0	0	2	0	1	5
DLPFC	2	0	0	0	2	0	1	5
IFC	2	0	0	0	2	0	1	5
iPFC	2	0	0	0	2	0	1	5
PCC	2	0	0	0	1	0	1	4
VLT	1	0	0	0	1	0	1	3
TempPole	1	0	0	0	1	0	0	2
MRI-Death duration (years; SD)	2.0 (1.26)	2.80 (1.92)	2.0 (0)	2.0 (0)	3.5 (2.12)	1.8 (0.94)	5.0 (0)	2.55 (1.54)

eTable 1: Number of pathologic burden samples per region per clinical phenotype, as well as MRI-to-death duration in years

eResults:

Results comparing BASIL CBF and BASIL CBF with PVC:

Results are similar for BASIL CBF with PVC, BASIL CBF without PVC, and CBF calculated using a simple algebraic mean.

Fixed Effect	*BASIL + PVC*	*BASIL*	*Simple mean*
MRItoDeath	**806.45**	**807.83**	**816.72**

Akaike information criteria (AIC) of cerebral blood flow (CBF) estimates for linear mixed-effect models (LMMs). For each model, the natural log of the scaled percent area occupied was the dependent variable and the CBF estimate and MRI to death duration were fixed effects. Participant was included as a random effect and the duration between MRI and death was a fixed effect of no interest. All p-values by Vuong's closeness test for non-nested models $p>0.05$ (uncorrected for multiple comparisons)

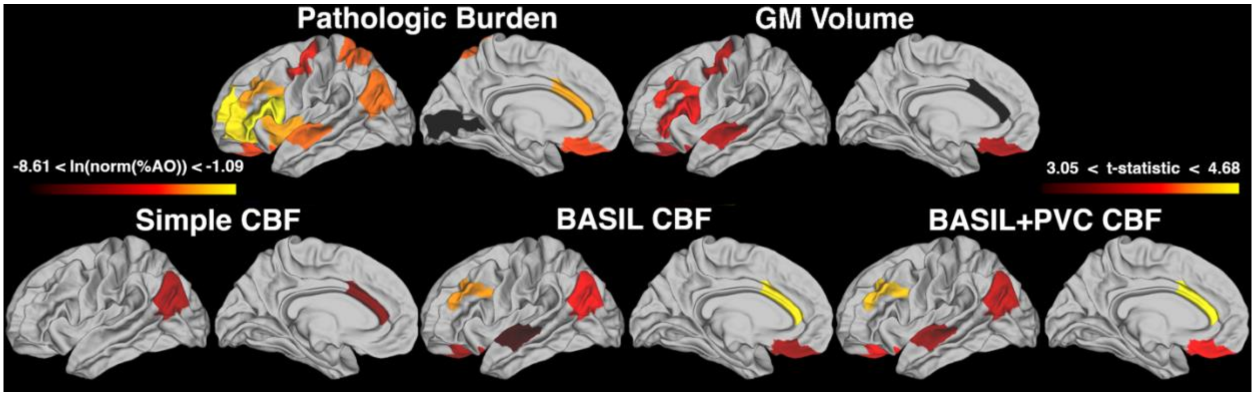

Comparison of cerebral blood flow (CBF) and gray matter (GM) volumes between patients with frontotemporal lobar degeneration due to tau (FTLD-tau) and controls. Mean postmortem pathologic burden of FTLD-tau inclusions (natural log of min-max normalized percent area occupied; ln(norm(%AO))) in all regions with > 4 samples are shown in the top left panel. The next panels are comparisons of *in vivo* MRI measures between patients with FTLD-tau and controls with differences considered significant at $p<0.05$, after Bonferroni correction for multiple comparisons, again in all regions with > 4 samples. Scale bars for CBF and volumes are t-statistics. Only regions reaching significance are shown.

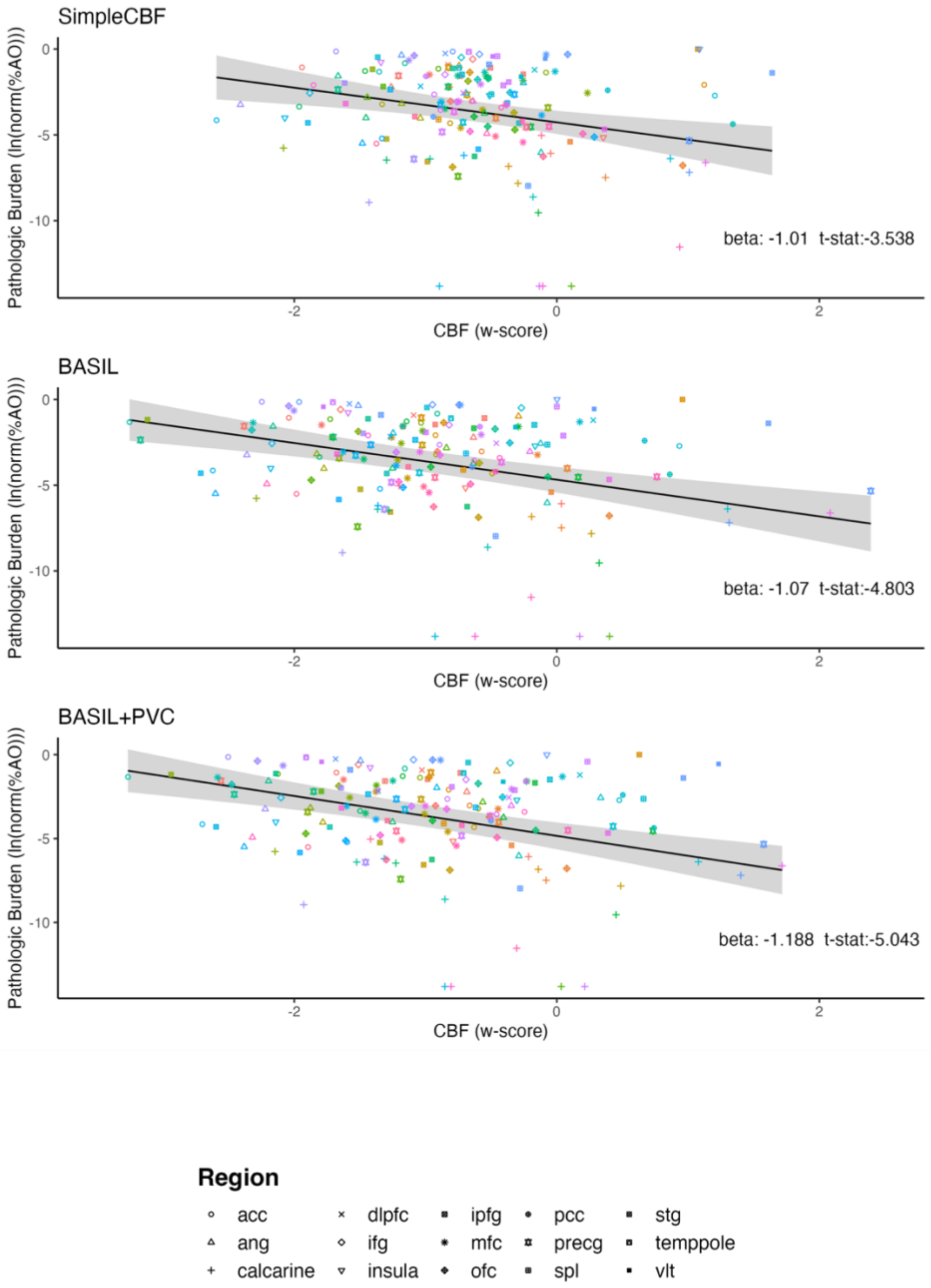

Cerebral blood flow (CBF) estimates and pathologic burden. Each cerebral blood flow (CBF) estimate has a significant relationship with pathologic burden (p<0.005, after Bonferroni correction for multiple comparisons). Each point represents measures from one region, and each color represents one participant. The trend lines represent best fits of the w-Scores of the CBF estimate as a fixed effect on the natural log of the percent area occupied data (ln(%AO)) in models with MRI-to-autopsy interval and participant as additional effects in the linear mixed-effects models, with the grey representing the 95% confidence intervals of the model fit. Betas and t-statistics are reported for the fixed effect of the CBF estimate. Shape of each point represents region (though this was not accounted for statistically). Abbreviations: anterior cingulate (ACC), superior/middle temporal gyrus (STG), orbitofrontal cortex (OFC), middle frontal cortex (MFC), calcarine cortex, angular gyrus (ANG), precentral gyrus (PrecG), dorsolateral

prefrontal cortex (DLPFC), inferior frontal gyrus (IFG), inferior parietal gyrus (IPFG), superior parietal lobe (SPL), insula, posterior cingulate cortex (PCC), temporal pole (temppole), and ventrolateral temporal cortex (VLT)

eReferences

Abraham, A., Pedregosa, F., Eickenberg, M., Gervais, P., Mueller, A., Kossaifi, J., Gramfort, A., Thirion, B., & Varoquaux, G. (2014). Machine learning for neuroimaging with scikit-learn. *Frontiers in Neuroinformatics*, *8*(FEB), 1–10. https://doi.org/10.3389/fninf.2014.00014

Adebimpe, A., Bertolero, M., Dolui, S., Cieslak, M., Murtha, K., Baller, E. B., Boeve, B., Boxer, A., Butler, E. R., Cook, P., Colcombe, S., Covitz, S., Davatzikos, C., Davila, D. G., Elliott, M. A., Flounders, M. W., Franco, A. R., Gur, R. E., Gur, R. C., … Satterthwaite, T. D. (2022). ASLPrep: a platform for processing of arterial spin labeled MRI and quantification of regional brain perfusion. *Nature Methods*, *19*(6), 683–686. https://doi.org/10.1038/s41592-022-01458-7

Alsop, D. C., Detre, J. A., Golay, X., Gunther, M., Hendrikse, J., Hernandez-Garcia, L., Lu, H., Macintosh, B. J., Parkes, L. M., Smits, M., Van Osch, M. J. P., Wang, D. J. J., Wong, E. C., & Zaharchuk, G. (2015). Recommended implementation of arterial spin-labeled Perfusion mri for clinical applications: A consensus of the ISMRM Perfusion Study group and the European consortium for ASL in dementia. *Magnetic Resonance in Medicine*, *73*(1), 102–116. https://doi.org/10.1002/mrm.25197

Burke, S. E., Phillips, J. S., Olm, C. A., Peterson, C. S., Cook, P. A., Gee, J. C., Lee, E. B., Trojanowski, J. Q., Massimo, L., Irwin, D. J., & Grossman, M. (2022). Phases of Volume Loss in Patients With Known Frontotemporal Lobar Degeneration Spectrum Pathology. *Neurobiology of Aging*, *113*, 95–107. https://doi.org/10.1016/j.neurobiolaging.2022.02.007

Buxton, R. B., Frank, L. R., Wong, E. C., Siewert, B., Warach, S., & Edelman, R. R. (1998). A general kinetic model for quantitative perfusion imaging with arterial spin labeling. *Magnetic Resonance in Medicine*, *40*(3), 383–396. https://doi.org/10.1002/mrm.1910400308

Chappell, M. A., Groves, A. R., MacIntosh, B. J., Donahue, M. J., Jezzard, P., & Woolrich, M. W. (2011). Partial volume correction of multiple inversion time arterial spin labeling MRI data. *Magnetic Resonance in Medicine*, *65*(4), 1173–1183. https://doi.org/10.1002/mrm.22641

Chappell, M. A., Groves, A. R., Whitcher, B., & Woolrich, M. W. (2009). Variational Bayesian inference for a nonlinear forward model. *IEEE Transactions on Signal Processing*, *57*(1), 223–236. https://doi.org/10.1109/TSP.2008.2005752

Cox, R. W., & Hyde, J. S. (1997). Software tools for analysis and visualization of fMRI data. *NMR in Biomedicine*, *10*(4–5), 171–178. https://doi.org/10.1002/(SICI)1099-1492(199706/08)10:4/5<171::AID-NBM453>3.0.CO;2-L

Gorgolewski, K., Burns, C. D., Madison, C., Clark, D., Halchenko, Y. O., Waskom, M. L., & Ghosh, S. S. (2011). Nipype: A flexible, lightweight and extensible neuroimaging data processing framework in Python. *Frontiers in Neuroinformatics*, *5*(August). https://doi.org/10.3389/fninf.2011.00013

Harris, C. R., Millman, K. J., van der Walt, S. J., Gommers, R., Virtanen, P., Cournapeau, D., Wieser, E., Taylor, J., Berg, S., Smith, N. J., Kern, R., Picus, M., Hoyer, S., van Kerkwijk, M. H., Brett, M., Haldane, A., del Río, J. F., Wiebe, M., Peterson, P., … Oliphant, T. E. (2020). Array programming with NumPy. *Nature*, *585*(7825), 357–362. https://doi.org/10.1038/s41586-020-2649-2

Jenkinson, M., Bannister, P., Brady, M., & Smith, S. (2002). Improved Optimization for the Robust and Accurate Linear Registration and Motion Correction of Brain Images. *NeuroImage*, *17*(2), 825–841. https://doi.org/10.1006/nimg.2002.1132

Jenkinson, M., & Smith, S. (2001). A global optimisation method for robust affine registration of brain images. *Medical Image Analysis*, *5*(2), 143–156. https://doi.org/10.1016/S1361-8415(01)00036-6

Lanczos, C. (1964). Evaluation of Noisy Data. *Journal of the Society for Industrial and Applied Mathematics Series B Numerical Analysis*, *1*(1), 76–85. https://doi.org/10.1137/0701007

Power, J. D., Mitra, A., Laumann, T. O., Snyder, A. Z., Schlaggar, B. L., & Petersen, S. E. (2014). Methods to detect, characterize, and remove motion artifact in resting state fMRI. *NeuroImage*, *84*. https://doi.org/10.1016/j.neuroimage.2013.08.048

Virtanen, P., Gommers, R., Oliphant, T. E., Haberland, M., Reddy, T., Cournapeau, D., Burovski, E., Peterson, P., Weckesser, W., Bright, J., van der Walt, S. J., Brett, M., Wilson, J., Millman, K. J., Mayorov, N., Nelson, A. R. J., Jones, E., Kern, R., Larson, E., … Vázquez-Baeza, Y. (2020). SciPy 1.0: fundamental algorithms for scientific computing in Python. *Nature Methods*, *17*(3), 261–272. https://doi.org/10.1038/s41592-019-0686-2
